# Potential Correlates of Internet Gaming Disorder Among Indonesian Medical Students: Cross-sectional Study

**DOI:** 10.2196/25468

**Published:** 2021-04-19

**Authors:** Kristiana Siste, Enjeline Hanafi, Lee Thung Sen, Petra Octavian Perdana Wahjoepramono, Andree Kurniawan, Ryan Yudistiro

**Affiliations:** 1 Department of Psychiatry Faculty of Medicine Universitas Indonesia - Dr Cipto Mangunkusumo General Hospital Jakarta Indonesia; 2 Faculty of Medicine Universitas Pelita Harapan Siloam Hospitals Tangerang Indonesia; 3 Department of Internal Medicine Faculty of Medicine Universitas Pelita Harapan Siloam Hospital Tangerang Indonesia

**Keywords:** internet gaming disorder, medical students, psychopathology, temperament, risk factors

## Abstract

**Background:**

Internet gaming disorder has been a controversial topic for nearly a decade. Although internet addiction has been studied in medical students, there is a paucity of evidence regarding internet gaming disorder. Previous studies in Indonesia explored only the prevalence rate and characteristics.

**Objective:**

This study aimed to determine the prevalence rate of internet gaming disorder and correlations between internet gaming disorder, temperament, and psychopathology among Indonesian medical students.

**Methods:**

A cross-sectional study was performed from August 2019 to September 2019 using total and convenience sampling at a private university and a public university, respectively. The study variables were measured using the Indonesian version of the 10-item Internet Gaming Disorder Test, the Temperament and Character Inventory, and the Symptoms Checklist 90. Chi-square and logistic regression analyses were conducted to examine the relationships between demographic factors, temperament, psychopathology, and the presence of internet gaming disorder.

**Results:**

Among the 639 respondents, the prevalence rate of internet gaming disorder was 2.03% (n=13), with a mean age of 20.23 (SD 0.13) years and an average gaming duration of 19.0 (SD 0.96) hours/week. Up to 71.2% respondents played using their mobile phones, and respondents with internet gaming disorder reported experiencing all psychopathologies assessed, except phobic anxiety. Bivariate analysis demonstrated that internet gaming disorder was associated with gender, gaming duration, gaming community affiliation, and 9 out of 10 domains of psychopathology. In a logistic regression model, internet gaming disorder was correlated with weekly gaming hours ≥20 hours (odds ratio [OR] 4.21, 95% CI 1.08-16.38, *P*=.04).

**Conclusions:**

These findings suggest that the prevalence of internet gaming disorder among medical students in Jakarta, Indonesia is similar to that in other populations of Asian countries. The predisposing factor for internet gaming disorder was weekly gaming duration, while other demographic, temperament, and psychopathology variables acted as probable moderators. Strategies should, therefore, be developed and integrated into medical curriculum to screen and aid individuals with these predisposing factors.

## Introduction

In this globalized era, the internet has become a necessity for people. It offers many benefits, especially for gamers, by connecting them digitally with others worldwide. Unfortunately, the internet bears its own negative effects when used excessively, which may lead to internet gaming disorder. Between 1995 and 2015, the percentage of internet users escalated (from 0%-14% to 45%-99% in 29 countries [1]). For internet gaming disorder, the prevalence globally varies from 0.7% to 15.6% among adults [2] and ranged from 0.6% to 19.9% among adolescents [3]. Specifically, a study in the US noted 8.5% of surveyed teenagers fulfilled criteria of internet gaming disorder [4], while German researchers revealed a prevalence of 1.2% [5]. Among Asian adolescents, a Taiwanese study discovered an internet gaming disorder rate of 3.1% [6], and a Japanese study demonstrated a prevalence 1.8% [7].

In Indonesia, internet usage rose from 0.9% in 2000 to 17.1% in 2014. Approximately 80% of the teenagers in Indonesia are on the internet daily [8]; consequently, the rate of internet gaming disorder among Indonesian teenagers is estimated at 10.15% [9] A systematic review [10] noted the high pooled prevalence of internet addiction (30.1%) among medical students [10], which is not surprising as this is a highly stressed group who are vulnerable to psychopathologies [11]. As such, they are at risk of adopting passive coping mechanisms. However, limited studies [10] have reported the more specific form of internet addiction (ie, internet gaming disorder) among medical students.

The varying prevalence of internet gaming disorder is due to the absence of consensus in diagnosing internet gaming disorder. Past studies [12] have looked at internet addiction as a generalized construct which encompasses all digital activities, and others, such as internet gaming, as a specific entity; however, the Diagnostic and Statistical Manual of Mental Disorders Fifth edition (DSM-5) and International Classification of Diseases 11 (ICD-11) have provided guidelines to identify affected individuals. According to the DSM-5, internet gaming disorder is defined as the persistent and recurrent use of the internet to play games that causes clinically significant impairment or distress. The patient must exhibit at least 5 out of 9 diagnostic criteria—preoccupation, withdrawal, tolerance, loss of control, giving up on other activities, continuation, deception, escape, and negative consequences—within a 12-month period [13]. Meanwhile, ICD-11 describes gaming disorder as a persistent or recurrent gaming behavior, either online or offline. The ICD-11 mentions 3 symptoms of gaming disorder, namely, impaired control over gaming, gaming being prioritized over daily activities and life interests, and its continuation or escalation, even after a negative impact has arisen [14]. Both internet gaming disorder (DSM-5) and internet gaming disorder (ICD-11) cover offline and online games [13], and accumulating evidence since the inclusion of internet gaming disorder within the section 3 of DSM-5 has led to international consensus for formal categorization of the disorder within ICD-11, which includes the internet as a specific identifier of the disorder [15]. As research and understanding of the disorders across populations grow, the nosology will require updates [15].

Researchers have developed numerous instruments to screen individuals afflicted with internet gaming disorder; however, there is no agreement regarding either screening tools or which clinical guidelines to adopt while diagnosing internet gaming disorder because there are limited studies on the diagnostic pathway, terminology definitions are arguable, and the diagnostic category is rather novel [16]. There are no definite indications yet for internet gaming disorder screening, and further research is required to define the risk and protective factors in order to narrow the specific population appropriate for screening [17].

Several studies in Indonesia have already described the prevalence, characteristics, and impacts of internet gaming disorder [9,18]. However, none of these studies focused on the relationship between internet gaming disorder and temperament, and internet gaming disorder and psychopathology. The main purpose of this study, hence, was to assess the correlations between internet gaming disorder, temperament, and psychopathology among medical students in Indonesia, given the prevalence of internet gaming disorder among Indonesian young adults and thus allowing for the ratification of preventive modules in medical schools.

## Methods

### Design

A cross-sectional study was conducted in private and public universities in Jakarta. The capital city of Indonesia was believed to be a representative sample base due to its dense and diverse population from across the archipelago. Total sampling in the private university and convenience sampling in the public university were performed from August to September 2019. This study was approved by Mochtar Riady Institute for Nanotechnology Ethics Committee (protocol number 1906010-02). Written consent was obtained from each respondent. Respondents who were screened with internet gaming disorder or other psychopathologies were invited for further psychiatric therapy sessions.

### Respondents

The target population of this study was medical students in Indonesia, represented by the medical students in Jakarta. The sample consisted of medical students from private and public universities who had fulfilled the inclusion from respective and exclusion criteria (absence of severe psychotic disorders and substance use) by means of self-report and brief semistructured interviews with the research team. Criteria were chosen to ensure that the associations that generated were specific to internet gaming disorder. The respondents were briefed about the study in a specific session; respondents were required to give informed consent in order to participate. The minimal sample size was tabulated using the cross-sectional and hypothesis confirmation studies formula, with a type I error of 1.96, type II error of 0.84, and an absolute precision of .05 [19].

### Measurements

The respondents completed a questionnaire providing demographic data and gaming-related characteristics (duration, affiliation to gaming community, and genre, whereby examples were provided). The game genres, which were adapted from prior studies [20-22], were divided into massive multiplayer role-playing games, multiplayer online battle arena, first-person shooting, multiplayer battle royale, real-time strategy, turn-based strategy, simulation, puzzle, or music/sports/platform. In addition, respondents answered 3 self-rated questionnaires.

The Indonesian version of the 10-item Internet Gaming Disorder Test (IGDT-10) consists of 10 statements about online gaming disorder similar to those of the original version. A 3-point Likert scale was used for response options (never, sometimes, and often). Scoring was then dichotomized (often=1; never or sometimes=0) to mirror the dichotomous nature of DSM-5 criteria. Items 9 and 10 represented the same construct and thus only a single score was used (ie, a response of “often” on either item scored only 1 point). The cut-off for internet gaming disorder, in accordance with DSM-5 criteria, specified a minimum of 5 out of 9 criteria [23]. In this study, we adopted the definition “persistent and recurrent use of the Internet to engage in games, often with other players, leading to clinically significant impairment or distress” for internet gaming disorder, and despite the terminology, internet gaming disorder encompassed individuals exhibiting problematic gaming symptoms in both offline and online settings [24]. For succinctness, respondents meeting the IGDT-10 cut-off were classified herein as having internet gaming disorder as IGDT-10 was found to cover clinical criteria of both DSM-5 and ICD-11 [25], though it should be cautioned that the instrument is a screening tool.

The Indonesian version of the modified Temperament and Character Inventory (TCI) was used to measure temperament. The questionnaire consists of 23 questions assessing the 3 domains of temperament (novelty seeking, reward dependence, and harm avoidance), and 16 questions that assess character (self-directedness and self-transcendence). However, this study only employed the former, and the partial instrument reliability was 52%. The answers were either “yes” (2 points) or “no” (1 point), and the points were summed up according to each domain. Each domain was divided into “low,” if the total score was lower than average, and “high,” if the total score was higher than average [26,27].

The Indonesian version of Symptoms Checklist 90 (SCL-90) has 82.9% sensitivity and 83.0% specificity. Respondents were asked if they had experienced any of the symptoms (90 statements) during the previous 7 days (1 week) using a 5-point Likert scale (0=never and 4=always). The points were summed based on somatization, obsessive-compulsiveness, interpersonal sensitivity, depression, anxiety, hostility, phobic anxiety, paranoid ideation, psychoticism, and additional symptoms. Respondents were then divided into a *No* group if the total score was <61, and a *Yes* group if the total T-score was ≥61 [28,29].

### Statistical Analysis

The data were analyzed using statistical software (Statistical Package for Social Sciences for Windows version 25, IBM Corp). The characteristics of respondents are reported descriptively. The relationships of characteristics, temperament, and psychopathology with internet gaming disorder were determined using chi-square and Fisher exact tests. Furthermore, stepwise binomial logistic regression analysis was applied to examine the multivariate relationship between internet gaming disorder, the respondents’ characteristics, their temperaments, and psychopathologies (mean raw score and standard deviation are presented). Nine variables were chosen between respondents’ characteristics and SCL -90, results of the TCI were not included as none were significant; the criteria set was *P*<.25 [30] and relevance was based on prior studies [31,32] in the field.

## Results

### Respondent Characteristics

In total, there were 639 respondents (mean age 19.9 years), of whom 59.6% were late adolescents (381/639); 64.2% were female (410/639; mean age 19.7 years), and 35.8% were male (229/639; mean age 20.2 years). Respondents started gaming at a mean age of 11.31 years (SD 0.14) and had mean weekly gaming duration of 15.34 hours (SD 0.57; male: mean 19.00 hours, SD 0.96; female: mean 13.29 hours, SD 0.69); 55.2% of the respondents (353/639) lived independently, and 85.8% played games before 8 years of age (548/639). The age cut-off was based on a previous study [33]. Twice as many males (75/229, 32.8%) played more than 20 hours a week compared to females (68/410, 16.6%). Approximately 22.3% of males (51/229) joined a gaming community, compared to 12.0% of females (49/410). Device-wise, both genders preferred smartphones for playing games (455/639, 71.2%) (Table 1).

**Table 1:** Respondent demographics.

Characteristic		Male (n=229), n (%)	Female (n=410), n (%)	All (N=639), n (%)
**Age**				
	Early adolescence	0 (0.0)	1 (0.2)	1 (0.2)
	Mid-adolescence	10 (4.4)	39 (9.5)	49 (7.7)
	Late adolescence	130 (56.8)	251 (61.2)	381 (59.6)
	Adult	89 (38.9)	119 (29.0)	208 (32.6)
**Residence**				
	Independent	114 (49.8)	239 (58.3)	353 (55.2)
	Parent's house	115 (50.2)	171 (41.7)	286 (44.8)
**Gaming onset age**				
	≤8 years	187 (81.7)	361 (88.0)	548 (85.8)
	>8 years	42 (18.3)	49 (12.0)	91 (14.2)
**Time spent gaming**				
	≤20 hours/week	154 (67.2)	342 (83.4)	496 (77.6)
	>20 hours/week	75 (32.8)	68 (16.6)	143 (22.4)
**Gaming community affiliation**				
	Yes	51 (22.3)	49 (12.0)	100 (15.7)
	No	178 (77.7)	361 (88.0)	539 (84.4)
**Device**				
	Smartphone	122 (53.3)	333 (81.2)	455 (71.2)
	PC/desktop	41 (17.9)	25 (6.1)	66 (10.3)
	Laptop	55 (24.0)	38 (9.3)	93 (14.6)
	Tablet	11 (4.8)	14 (3.4)	25 (3.9)
**Internet gaming disorder**				
	Yes	9 (3.9)	4 (1.0)	13 (2.0)
	No	220 (96.1)	406 (99.0)	626 (98.0)

### Respondents With Internet Gaming Disorder

Of the 639 respondents, 13 respondents were identified with internet gaming disorder (2.03%). The 13 respondents on average scored 5.56 (SD 0.53) on IGDT-10, compared to a mean score of 0.37 (SD 0.76) among the rest of the respondents. There was a statistically significant difference between gender, with males being 4 times more likely than females to have internet gaming disorder (odds ratio [OR] 4.15, 95% CI 1.26-13.64; *P*=.02). In addition, respondents playing >20 hours per week were 6 times more likely to be screened with internet gaming disorder (OR 5.82, 95% CI 1.87-18.08; *P*=.002). Attachment to a gaming community also displayed a statistical significance (OR 8.08, 95% CI 2.12-30.81; *P*=.04). No significant relationship was observed for age (*P*=.74), onset of gaming (*P*>.99), type of residence (*P*=.51), and device of choice (*P*=.57) with gaming disorder (Table 2). Among respondents with internet gaming disorder, 6 respondents (46.2%) mainly played multiplayer online battle royale, 4 respondents (30.8%) primarily enjoyed multiplayer online battle arena, 2 respondents (15.4%) focused on puzzle games, and 1 respondent (7.7%) chiefly played turn-based strategy games. Among respondents without internet gaming disorder, the most popular game genre was multiplayer online battle arena (190/626, 30.4%), second-most popular was multiplayer online battle royale (118/626, 18.9%), followed by real-time strategy (72/626, 11.5%), puzzle genre (69/626, 11.0%), and simulation (33/626, 5.3%).

**Table 2:** Comparison of demographics between respondents with and without internet gaming disorder.

Category		Internet gaming disorder			Comparison		
		Yes, n		No, n	Chi-square (*df*)	*P* value	OR^a^ (95% CI)
Total		13	626				
**Age**					1.257 (3)	.74	—^b^
	Early adolescence	0		1			
	Mid-adolescence	0		49			
	Late adolescence	9		372			
	Adult	4		204			
**Gender**					6.435 (1)	.02	4.15 (0.13-13.64)
	Male	9		220			
	Female	4		406			
**Gaming onset age**					0.014 (1)	>.99	1.10 (0.24-5.03)
	≤8 years	2		89			
	>8 years	11		537			
**Time spent gaming**					11.715 (1)	.002	5.82 (1.87-18.08)
	≤20 hours/week	5		491			
	>20 hours/week	8		135			
**Residence**					0.443 (1)	.51	1.45 (0.48-4.37)
	Independent	6		347			
	Parent's house	7		279			
**Community**					5.231 (1)	.04	8.08 (2.12-30.81)
	Yes	5		95			
	No	8		531			
**Device**					2.037 (3)	.57	—
	Smartphone	7		448			
	PC/desktop	2		64			
	Laptop	3		90			
	Tablet	1		24			

^a^OR: odds ratio.

^b^Value missing as chi-square analysis would not produce risk estimate for *df* >1.

### TCI and Internet Gaming Disorder

Of the 3 TCI domains, none was found to have a significant relationship with internet gaming disorder (novelty seeking: *P*=.13; reward dependence: *P*=.35; harm avoidance: *P*=.18). Eight out of 13 respondents with internet gaming disorder exhibited high novelty seeking behavior; similarly, 67% of respondents (10/13) also had high harm avoidance, and approximately only 33% of respondents (2/13) displayed high reward dependence (Table 3).

**Table 3:** Associations between Temperament and Character Inventory and internet gaming disorder.

Temperament and Character Inventory		Internet gaming disorder			Comparison		
		Yes	No	Chi-square (*df*)		*P* value	OR^a^ (95% CI)
**Novelty seeking**				2.276 (1)		.13	2.33 (0.75-7.20)
	High	8	255				
	Low	5	371				
**Reward dependence**				0.56 (1)		.35	1.78 (0.39-8.23)
	High	2	58				
	Low	11	568				
**Harm avoidance**				1.71 (1)		.18	0.43 (0.12-1.59)
	High	10	555				
	Low	3	71				

^a^OR: odds ratio.

### Association Between Internet Gaming Disorder and Psychopathology

The Symptoms Checklist 90 (SCL-90) has 9 domains with supplementary Global Symptoms Index (GSI) and an additional domain in the Indonesian version. Overall, respondents with internet gaming disorder scored 2 to 3 times higher on all domains of SCL-90 than respondents without internet gaming disorder. Out of all the domains, only phobic anxiety (*P*>.99) and the additional domain (*P*=.20) were not statistically significant (Table 4). GSI and interpersonal sensitivity were found to be markedly significant (OR 12.87, 95% CI 3.96-41.78, *P*<.001; OR 23.18, 95% CI 5.35-100.46, *P*=.001) respectively. Two out of 13 respondents (15%) who scored highly on IGDT-10 presented with positive somatization, obsessive-compulsive disorder, depression, anxiety, hostility, and paranoid symptoms. Interpersonal sensitivity and psychoticism were reported by 3 respondents. Those with low IGDT-10 scores also reported similar psychopathologies; for instance, 6 respondents without problematic gaming also had positive somatization symptoms. Phobic anxiety symptoms were exclusively reported by 7 respondents without internet gaming disorder.

**Table 4:** Symptoms Checklist 90 and internet gaming disorder.

Psychopathology			Internet gaming disorder								Comparison				
			Yes				No				Chi-square (*df*)		*P* value		OR^a^ (95% CI)
			n (%)		Mean (SD)		n (%)		Mean (SD)						
**Global Symptoms Index**					150.1 (72.3)				54.9 (54.1)		28.93 (1)		<.001		12.87 (3.96-41.78)
	Yes	5 (0.8)				29 (4.5)									
	No	8 (1.3)				597 (93.4)									
**Somatization**					14.3 (11.0)				5.9 (6.9)		21.44 (1)		.01		18.79 (3.41-103.64)
	Yes	2 (0.3)				6 (0.9)									
	No	11 (1.7)				620 (97.0)									
**Obsessive compulsiveness**					23.1 (10.0)				8.7 (7.9)		21.44 (1)		.01		18.79 (3.41-103.64)
	Yes	2 (0.3)				6 (0.9)									
	No	11 (1.7)				620 (97.0)									
**Interpersonal sensitivity**					20.3 (10.3)				7.0 (7.0)		35.77 (1)		.001		23.18 (5.35-100.46)
	Yes	3 (0.5)				8 (1.3)									
	No	10 (1.6)				618 (96.7)									
**Depression**					26.4 (12.6)				9.3 (9.6)		21.44 (1)		.01		18.79 (3.41-103.64)
	Yes	2 (0.3)				6 (0.9)									
	No	11 (1.7)				620 (97.0)									
**Anxiety**					10.5 (8.0)				3.5 (4.6)		10.78 (1)		.03		9.30 (1.86-46.60)
	Yes	2 (0.3)				12 (1.9)									
	No	11 (1.7)				614 (96.1)									
**Hostility**					8.1 (4.5)				3.1 (3.7)		13.14 (1)		.02		11.20 (2.19-57.22)
	Yes	2 (0.3)				10 (1.6)									
	No	11 (1.7)				616 (96.4)									
**Phobic anxiety**					7.5 (5.1)				3.2 (4.0)		0.15 (1)		>.99		0.98 (0.97-0.99)
	Yes	0 (0)				7 (1.1)									
	No	13 (2.0)				619 (96.9)									
**Paranoid ideation**					11.2 (6.6)				4.1 (4.5)		10.78 (1)		.03		9.30 (1.86-46.60)
	Yes	2 (0.3)				12 (1.9)									
	No	11 (1.7)				614 (96.1)									
**Psychoticism**					10.3 (11.7)				5.2 (6.1)		35.77 (1)		.001		23.18 (5.35-100.46)
	Yes	3 (0.5)				8 (1.3)									
	No	10 (1.6)				618 (96.7)									
**Additional**					11.4 (5.2)				4.9 (4.9)		2.80 (1)		.20		5.13 (0.61-43.35)
	Yes	1 (0.1)				10 (1.6)									
	No	12 (1.9)				616 (96.4)									

^a^OR: odds ratio.

### Multivariate Analysis of Internet Gaming Disorder, Respondent Characteristics, TCI, and SCL-90

Logistic regression analysis was performed to identify the factors that influenced risk of internet gaming disorder. The results are shown in Table 5. In the first model, when controlling for gender and community affiliation, weekly gaming duration of >20 hours per week remained statistically significant (OR 3.98, 95% CI 1.20-13.18, *P*=.02). The model explained 12.1% of the variance (*R*^2^=0.121) and had acceptable goodness-of-fit with the Hosmer-Lemeshow test *P*=.74. When controlling for temperament and psychopathology, weekly gaming duration remained a significant factor associated with internet gaming disorder (OR 4.21, 95% CI 1.08-16.38, *P*=.04). The overall model improved with pseudo *R*^2^=0.301 and Hosmer-Lemeshow test *P*=.80.

**Table 5:** Logistic regression analysis of demographics, temperament, and psychopathologies with internet gaming disorder (dependent variable).

Variable	Model 1^a^				Model 2^b^		
	B	OR^c^ (95% CI)	*P* value	B		OR (95% CI)	*P* value
Constant	–5.17	N/A^d^	<.001	–5.18		N/A	<.001
Gender	1.07	2.93 (0.86-9.95)	.08	1.38		3.99 (0.92-17.34)	.06
Community	0.67	1.95 (0.59-6.49)	.28	0.32		1.38 (0.34-5.50)	.65
Time spent gaming	1.38	3.98 (1.20-13.18)	.02	1.44		4.21 (1.08-16.38)	.04
Novelty seeking	N/A	N/A	N/A	0.60		1.82 (0.46-7.18)	.40
Harm avoidance	N/A	N/A	N/A	–1.14		0.32 (0.07-1.41)	.13
Global Symptoms Index	N/A	N/A	N/A	1.54		4.67 (0.55-39.40)	.16
Somatization	N/A	N/A	N/A	0.91		2.48 (0.15-42.29)	.53
Obsessive compulsiveness	N/A	N/A	N/A	2.05		7.77 (0.22-276.61)	.26
Interpersonal sensitivity	N/A	N/A	N/A	0.90		2.47 (0.034-180.71)	.68
Depression	N/A	N/A	N/A	0.49		1.62 (0.099-26.74)	.73
Anxiety	N/A	N/A	N/A	–1.05		0.35 (0.003-46.97)	.68
Hostility	N/A	N/A	N/A	0.54		1.71 (0.11-25.82)	.70
Paranoia	N/A	N/A	N/A	–1.52		0.22 (0.002-22.93)	.52
Psychoticism	N/A	N/A	N/A	2.47		11.85 (0.074- >999.999)	.34
Additional	N/A	N/A	N/A	–2.78		0.06 (0.001-2.98)	.16

^a^χ_3_^2^=14.13, *P*=.003; Nagelkerke *R*^2^=0.121 Hosmer-Lemeshow test *P*=.74.

^b^χ_15_^2^=35.64, *P*=.002; Nagelkerke *R*^2^=0.301; Hosmer-Lemeshow test *P*=.80.

^c^OR: odds ratio.

^d^N/A: not applicable.

## Discussion

### Prevalence and Demographics of Internet Gaming Disorder Among Medical Students

In a sample of Indonesian medical students, 2.03% (13/639) were suspected to suffer from internet gaming disorder, and a weekly gaming duration >20 hours was predictive of internet gaming disorder. Gender and participation in a gaming community did not increase the odds of internet gaming disorder. Subscales of the SCL-90, with the exception of phobic anxiety, were completed by respondents with internet gaming disorder, but none was found to increase susceptibility to internet gaming disorder in this study. The prevalence of internet gaming disorder has previously been found to range between 0.27% and 57.50% [34] and that of internet addiction has been found to range from 0.8% to 26.7% [35]. Notably, prior studies [5,36-41] indicated that internet addiction and video game addiction or problematic gaming prevalence dropped within the older age groups. Another study with young respondents (mean age 13 years), reported a higher prevalence of up to 4% [42] and up to 9% in primary school students [4]. Alternatively, a systematic review [43] estimated a pooled prevalence of 20.0% and 10.1% for internet addiction and internet gaming disorder, respectively, among the general population in Southeast Asia.

In addition, we found an association between gender and internet gaming disorder, with males constituting more than twice the number of respondents with internet gaming disorder than females, which was consistent with previous findings [36,39,40,44,45]. Furthermore, males played longer and were more likely to join a gaming community than females. In prior studies in pathological gambling, several theories have been suggested, such as differences in genetics and neurobiology between genders [46,47]. A functional magnetic resonance imaging study demonstrated that males exhibited amplified connectivity in their mesocorticolimbic pathway when playing video games compared to that exhibited by females [48]. This pathway is known for its pivotal role in reward assessment, motivated behavior, and cognitive regulation through dopaminergic modulation [49,50]. On a larger scope, gender differences have already been observed in multitudes of addictions, although many studies were culturally and politically biased [51,52]. A review [53] argued that although biological processes within the brain differ between males and females in certain areas, and there are variations in genetic expression, these are, nonetheless, further influenced at the phenotypic level by sociocultural factors and individual experiences. Gendered experiences, such as boys playing with robots and females with dolls, combined with sociocultural notions of gender-based activities, in which gambling and competitive activities are perceived to be masculine, result in male and females tending to adopt opposite coping and escape mechanisms [53].

### Internet Gaming Disorder and Gaming Characteristics Among Medical Students

Consistent with the findings of previous studies [5,45], the correlation between weekly hours spent gaming and internet gaming disorder was significant (*P*=.002), with approximately 22% of affected respondents spending more than 20 hours a week engaging in such activities. This relationship was maintained even after controlling for other significant sociodemographic factors, such as gender and community participation, temperament, and psychopathology (OR 4.21, 95% CI 1.08-13.68). A previous qualitative study [54] argued that although increased gaming time is associated with internet gaming disorder, it should not be considered a criterion for addiction when no negative consequence was observed. The study highlighted the essence of context when considering gaming time. This was revealed to be pertinent as the 2 cases identified demonstrated contrasting psychological and behavioral patterns, even though they both played over 14 hours a day [54]. Additionally, gamers may not accurately account for the duration spent on activities related to games, including strategizing, discussing, and fantasizing [55], as actual time spent gaming, thus camouflaged as seemingly short gaming duration. Another study [56] indicated that the effects of increased gaming time, particularly on weekdays, were more likely to develop into depressive, psychosomatic, and musculoskeletal symptoms. A probable explanation relates to the interaction with motive. Positive excessive gameplay might add to a person’s life, in contrast to excessive play as a result of negative motivations, such as escapism. The time spent in gaming reduces the availability of time during which to perform other essential tasks, such as physical socializing, schoolwork, or work; thus, gameplay duration is a pivotal determinant when put together with gameplay motives [56-58]. Alternatively, serious gaming provided complementary avenue of training and education [59] and novel opportunities to socialize with individuals, from near and far. These generated social capitals, which are greatly influenced by physical and social proximity (familiarity) [60], more often than not spurred further indulgence in game activities [61] yet translate poorly to offline social support or provision of deep affective relationships [62,63].

Our study revealed a significant association between affiliation to a gaming community and internet gaming disorder (*P*=.04). Online gaming is an inherently social activity, particularly with the advent of massively massive multiplayer role-playing games, multiplayer online battle arena, and multiplayer online battle royale [20]. The majority of respondents with internet gaming disorder in our study favored multiplayer online battle royale and multiplayer online battle arena to other genres. Multiplayer online battle royale, as a survival game combined with scavenging and combat, presents gamers with unique scenarios and dynamic and competitive gameplay in each round [20,64]. Similarly, the game genre multiplayer online battle arena was also prevalent as it motivates gamers through nonrepetitive game style, array of in-game ranks and items, and emphasis on teamwork and clan [20]. The social factors in playing multiplayer online battle arena included making new friends, chatting, and working in a team. Social elements, such as developing reputation and admiration from the gaming community, were a central driving factor in obtaining enjoyment in playing these games [65]. Gamers with internet gaming disorder may have used online games as a substitute for establishing real-world relationships, as increasing internet gaming disorder symptoms are associated with social anxiety, and social motivations are associated with gaming addiction [66-69]. According to the social compensation hypothesis, the fleeting sense of social interaction in online games and the sense of escape from the physical world facilitates greater engagement in games [66,69]. This particular relationship was not explored in this study.

### Temperament, Psychopathology, and Internet Gaming Disorder

Our study did not find an association between TCI domains and internet gaming disorder. This finding conformed with the results of a recent study [33], which argued that the 3 domains of TCI were neither protective nor risk factors for internet addiction. Individuals with high novelty-seeking supposedly modulate dopaminergic and heritable tendencies toward intense excitement, unpredictable and emotional behavior, and repeated exploratory activities in response to novelty [70]. The lack of association is partly explained as individuals with high novelty seeking are readily disinterested and lack persistence in doing a particular activity. The individuals then favor shifting their activities almost impulsively, and thus they are less likely to suffer internet addiction [33]. Our study showed a small distinction between high and low scores in all TCI domains. A previous study [71] in South Korea displayed a significant total score difference between problematic internet users and problematic drug users, although the subanalysis of the novelty seeking score difference was not statistically significant. The study [71] also showed an insignificant correlation between novelty seeking and internet gaming disorder, and a significant relationship with harm avoidance and reward dependence scores in the problematic internet user group, but not in the control group. This is in contrast with the findings of a similar study which proposed that novelty seeking and harm avoidance were strong predictors of internet gaming disorder compared to those for healthy individuals [72]. The contradictory evidence suggests that it is necessary to investigate personality factors more deeply, as novelty seeking, harm avoidance, and reward dependence have been found to be determined by a person’s personality profile and their impulsivity [71]. 

Bivariate analysis revealed a correlation between respondents with internet gaming disorder and nearly all psychopathologies in the Indonesian version of SCL-90, except for phobia and the additional domains. This pattern may be an indication of the nonspecificity of psychological distress or a variety of clinical profiles presented by internet gaming disorder respondents. However, gender (*P*=.06), gaming community (*P*=.65), temperament (novelty seeking: *P*=.40; harm avoidance: *P*=.13), and psychopathological factors (GSI: *P*=.16; somatization: *P*=.53; obsessive compulsiveness: *P*=.26; interpersonal sensitivity: *P*=.68; depression: *P*=.73; anxiety: *P*=.68; hostility: *P*=.70; paranoia: *P*=.52; psychoticism: *P*=.34; additional: *P*=.16) lost significance in multivariate analysis. Nonetheless, incorporating temperament and psychopathology enhanced the overall model pseudo *R*^2^ from 0.12 to 0.30, suggesting that they may be important moderators. The complex interaction between online video game overuse and associated psychopathologies has persisted and is apparent in the ambiguous evidence available. Several cross-sectional [67,73,74] and cohort studies [4] described significant effects associated with social phobia, gaming, and addiction, while other studies failed to establish any association [75-77].

Our study had limited identification of the dysfunctions experienced by respondents at a single point in time; however, the directional causality between internet gaming disorder and psychopathology is of high importance. An elaborate cohort study [4] demonstrated that individuals who engaged in chronic persistent gaming developed depression, anxiety, and social phobia after 2 years. In addition, they had significantly lower grades and social functioning compared to those who recovered from internet gaming disorder or who did not develop internet gaming disorder [4]. Based on the proposed Escape Theory [78], a number of studies have depicted gaming as an escape strategy for patients dealing with depression [68,79]. Nonetheless, it has been argued that gaming addiction should not be viewed as merely an escape or defective coping mechanism because symptoms of other disorders are also evident [4]. Internet gaming disorder might worsen psychopathological distress, while the management of internet gaming disorder could alleviate these complaints [4]. It is uncertain, however, as to whether the psychopathologies were preexisting and then reinforced by internet gaming disorder, as previous longitudinal research has failed to establish significant causality between internet gaming disorder and symptoms of depression and anxiety [77]. 

### Strengths and Limitations

This study is the first, to the knowledge of the authors, to investigate associations between temperament, psychopathologies, and internet gaming disorder in medical students in Indonesia. Additionally, our study was able to recruit a considerable number of respondents. Our study also distinguished online gaming by platform, from computers to mobile phones, given that an expanding number of people are occupied by digital games. Data from our study suggest that incorporating selective preventive measures among medical students targeted to proactively shift and shape gaming as positive and nurturing experiences within the medical education field is required. Prior research has demonstrated that serious games can be developed for the purpose of medical education and provided moderate effects in aiding transfer of knowledge or skills, with the additional benefit of generating a motivating and recreational learning experience [80]. Some have noted the lack of standard in pedagogical approaches of these game develops, but efforts are on-going to generate a structured framework [81] and advocate the use of serious games, which are disparate from commercial games, as prevention for gaming addiction [82].

One specific limitation of our study, apart from being cross-sectional, was that the samples were taken from medical students alone. Interpretations of correlations, or absence thereof, should be made with the sample scope in mind. Although the prevalence of internet gaming disorder among medical students fell within the range observed in the general population, the characteristics of the respondents differed largely from those of previous studies. For example, in our study, females comprised more than half of the respondents, whereas internet gaming disorder is more common in males, and past studies [35,39,75,83] have largely examined a male-dominant sample, This cross-sectional study utilized a convenience sample rather than randomized respondents, which introduced selection bias. Our study enrolled medical students to focus on an understudied population with respect to internet gaming disorder and which is presumed to have better health care access yet in reality most neglect to follow up on existing symptoms [10]; concurrently, the resources available limited how widespread sample recruitment could be. Further research should employ longitudinal design, wider population, randomized sampling, comparison to clinical psychiatric diagnoses, and investigate the development of potential interventions.

### Conclusions

Our study demonstrated that the point-prevalence of medical students screened with internet gaming disorder in Indonesia is within the estimated global range. The weekly duration of gaming was the strongest determinant of internet gaming disorder. Additional research should explore the diversity of motives and negative life consequences in engaged gamers to fully contextualize the effect of gaming duration and internet gaming disorder. Therefore, strategies should be developed and incorporated into medical curriculum to screen and aid students with prolonged gaming duration and psychopathologies.

## Abbreviations

DSM-5: Diagnostics and Statistical Manual for Mental Disorders, fifth edition
GSI: Global Symptoms Index
ICD-11: International Classification of Diseases, 11th revision
IGDT-10: 10-item Internet Gaming Disorder Test
OR: odds ratio
SCL-90: Symptoms Checklist 90
TCI: Temperament and Character Inventory
