# Correction: Potential Correlates of Internet Gaming Disorder Among Indonesian Medical Students: Cross-sectional Study

**DOI:** 10.2196/29790

**Published:** 2021-04-21

**Authors:** Kristiana Siste, Enjeline Hanafi, Lee Thung Sen, Petra Octavian Perdana Wahjoepramono, Andree Kurniawan, Ryan Yudistiro

**Affiliations:** 1 Department of Psychiatry Faculty of Medicine Universitas Indonesia - Dr Cipto Mangunkusumo General Hospital Jakarta Indonesia; 2 Faculty of Medicine Universitas Pelita Harapan Siloam Hospitals Tangerang Indonesia; 3 Department of Internal Medicine Faculty of Medicine Universitas Pelita Harapan Siloam Hospital Tangerang Indonesia

In “Potential Correlates of Internet Gaming Disorder Among Indonesian Medical Students: Cross-sectional Study” (J Med Internet Res 2021;23(4):e25468) the authors noted two errors.

Due to a system error, the name of one author, *Andree Kurniawan*, was replaced with the name of another author on the paper, *Ryan Yudistiro*. In the originally published paper, the order of authors was listed as follows:

Kristiana Siste; Enjeline Hanafi; Lee Thung Sen; Petra Octavian Perdana Wahjoepramono; Ryan Yudistiro; Ryan Yudistiro

This has been corrected to:

Kristiana Siste; Enjeline Hanafi; Lee Thung Sen; Petra Octavian Perdana Wahjoepramono; Andree Kurniawan; Ryan Yudistiro

In the originally published paper, the ORCID number of author *Ryan Yudistiro* was incorrectly published as follows:

0000-0002-5219-9029

This has been corrected to:

0000-0003-1418-2661

The correction will appear in the online version of the paper on the JMIR Publications website on April 21, 2021, together with the publication of this correction notice. Because this was made after submission to PubMed, PubMed Central, and other full-text repositories, the corrected article has also been resubmitted to those repositories.

